# Time From First Contact With Heart Team to Transcatheter Aortic Valve Replacement in the COVID-19 Era

**DOI:** 10.7759/cureus.41837

**Published:** 2023-07-13

**Authors:** Matthew J Billy, Zachary Brennan, Tariq Ahmad, John V Conte, Tyler J Wallen

**Affiliations:** 1 General Surgery, Geisinger Commonwealth School of Medicine, Scranton, USA; 2 Surgery, Michigan State University College of Osteopathic Medicine, East Lansing, USA; 3 Interventional Cardiology, Geisinger Commonwealth School of Medicine, Wilkes-Barre, USA; 4 Cardiothoracic Surgery, Geisinger Commonwealth School of Medicine, Wilkes-Barre, USA; 5 Cardiovascular Surgery, Geisinger Commonwealth School of Medicine, Wilkes-Barre, USA

**Keywords:** covid-19 in surgical patients, covid-19 delay of care, transcatheter aortic valve replacement, tavr, time to tavr

## Abstract

Objective: Transcatheter aortic valve replacement (TAVR) has become the dominant form of aortic valve replacement in the United States. During the Coronavirus disease 2019 (COVID-19) pandemic, access to elective surgical care was decreased, particularly for TAVR patients. In this study, we examine the impact of each COVID-19 “wave,” on our patient's access to TAVR procedures and their associated outcomes.

Methods: After institutional review board approval, we conducted a retrospective review of a prospectively maintained database and a review of our own center’s database to assess time to TAVR pre-COVID-19 and during internally defined COVID-19 “waves.” Statistical analysis was conducted via a t-test.

Results: We measured the time from first contact to TAVR and compared each COVID-19 wave to our institution's pre-COVID-19 data. During Wave 1 and 2 of COVID-19, our mean time to TAVR increased significantly to 68.44 ± 48.66 days (p = 0.05) and 68.94 ± 53.16 days (p = 0.02), respectively. All three COVID-19 waves demonstrated a statistically significant increase in all-cause mortality post-operatively (PO) with mean PO mortality of 2.5 (p = 0.0035), 1.33 (p = 0.0009), and 0.67 (p = 0.006), respectively, compared to pre-COVID-19 data.

Conclusions: Multiple studies have shown that increased time from first contact to TAVR results in increased morbidity and mortality. COVID-19 increased our institution's time to TAVR significantly across two waves with an increase in all-cause mortality in each wave. This study highlights the importance that institutions should develop mechanisms to ensure access to care during crises so that patients do not face potentially avoidable harm.

## Introduction

Transcatheter aortic valve replacement (TAVR) has become the dominant form of aortic valve replacement over the past decade for patients with aortic stenosis [1-4]. Previously, surgical aortic valve replacement (SAVR) was the treatment of choice for the majority of patients except for those at extreme risk [2-5]. Since 2019, TAVR has been extended to a much larger patient population, including those at low risk [4,5]. It is estimated that >350,000 procedures have been performed in >70 countries [6]. This growth has required a corresponding expansion in services and capacity, and, in some instances, this increased demand has overwhelmed a given system's capacity. In this scenario, wait times were frequently prolonged, which negatively impacted outcomes. Wijeysundera and colleagues demonstrated that a wait time of >60 days effectively negated the benefit of TAVR as compared to surgical valve replacement [7]. Further, multiple other studies have demonstrated that increased wait times were associated with greater mortality, hospitalization, and decline in both functional status and quality of life [8-10].

The COVID-19 pandemic fundamentally changed surgical practice and access to surgical care across the country and internationally. Many hospitals eliminated elective surgical cases, limited access to hospital beds, and reduced the number of working staff via cohorting practices. These practices impacted many patients, including those with aortic stenosis who required TAVR [11,12]. More specifically, however, each wave of COVID-19 presented its own unique set of challenges for our healthcare systems to overcome as policies frequently changed after the availability of a vaccine, a better understanding of COVID-19’s natural course and increased attention to patients who delayed or avoided care due to the pandemic. There are few studies and little data that identify how each wave of COVID-19 within the global health crisis affected patient access to care, especially in terms of cardiac disease. 

Thus, the present study aims to evaluate how the COVID-19 pandemic, broken down into each unique wave, affected patients' access to TAVR as compared to the pre-COVID-19 era and how that ultimately affected patient outcomes. 

## Materials and methods

Institutional review board approval was obtained in accordance with our surgical intuitional guidelines. This study was a retrospective review of a prospectively maintained database within a single institution, with patient data from two hospital facilities within the institution. To obtain patient data, we used a combination of access to our institution's Electronic Medical Healthrecord (EMR), The Society of Thoracic Surgeon (STS) database, and our own prospectively maintained TAVR database. Inclusion criteria included any patient undergoing TAVR from January 2019 until January 2022. This encompassed both pre-COVID-19 data and COVID-19-era data. Within the COVID-19-era data, we stratified patients into three regionally defined waves of the COVID-19 pandemic by surges and variants within our system’s patient population. We defined the “pre-COVID-19” time frame as the 1st quarter of 2019 to the 1st quarter of 2020. The first wave of COVID-19 was defined as the time from the 1st quarter of 2020 to the 2nd quarter of 2020. The second wave was defined as the 4th quarter of 2020 to 2nd quarter of 2021 and corresponded to the Delta variant. The 3rd wave was defined as the time between the 3rd quarter of 2021 and the 1st quarter of 2022. This third wave corresponded to the Omicron variant seen in our local population. 

There were no specific exclusion criteria, and all patients were included in this study. The primary outcome was defined as the time to TAVR, which was calculated as the time from the date of first contact with the heart team until the patient underwent TAVR, measured in days. The secondary outcome was all-cause mortality. 

Descriptive statistics were presented as the mean ± standard deviation (SD) for continuous variables, and percentage for categorical variables. The entire pandemic, and each wave, was compared to pre-COVID-19 data using unpaired t-test to obtain two-tailed p-values, with a threshold for significance of p < 0.05. 

## Results

**Figure 1:**
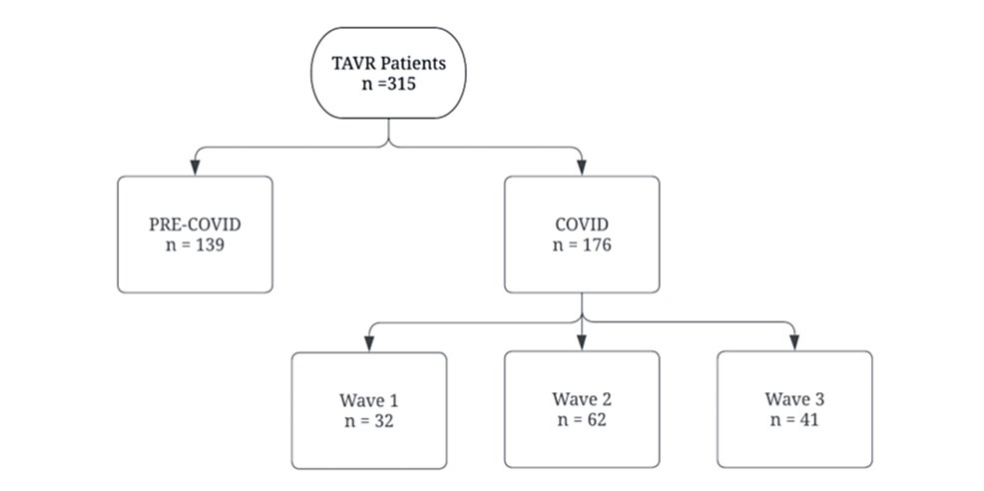
Total patients included in our study and stratified into each group TAVR: Transcatheter aortic valve replacement

We identified 315 patients who met the inclusion criteria. One-hundred and thirty-nine patients were included in the pre-COVID-19 arm from Quarter 1 (Q1) 2019 to Q1 2020. There were 176 patients in the COVID-19 arm that was subdivided into three waves as defined by our system given the presence of the SARS-CoV-2 virus and its variants. Wave 1 (Q1 2020 to Q2 2020) included 32 patients, Wave 2 [Delta variant] (Q4 2020 to Q2 2021) included 62 patients, and Wave 3 [Omicron variant] (Q3 2021 to Q1 2022) included 41 patients (Figure 1). 

In the pre-COVID-19 era, the mean time from first contact with the heart team to TAVR was 54.73 ± 31.76 days. During Wave 1, the mean time to TAVR significantly increased to 68.44 ± 48.66 days from first contact to TAVR (p= 0.05). During Wave 2, the mean time to TAVR significantly increased to 68.94 ± 53.16 days from first contact to TAVR (p= 0.019). During Wave 3, the mean time to TAVR increased to 53.56 ± 45.32 days from first contact to TAVR (p= 0.85) (Table 1).

**Table 1:** Mean time to TAVR from first contact stratified by each COVID wave and the pre-COVID timepoint FC = First contact with our institution's heart team TAVR: Transcatheter aortic valve replacement SD = Standard deviation CI = Confidence interval

COVID	n	Mean FC to TAVR (Mean days ± SD)	Min (days)	Max (days)	Range (days)	P-value	95% CI (days)
Pre-COVID	139	54.73 ± 31.76	3	216	3-216		
Wave 1	32	68.44 ± 48.66	4	192	4-192	0.05	-27.44 to 0.019 (-13.71)
Wave 2	62	68.94 ± 53.16	3	312	3-312	0.02	-26.13- -2.3 (-14.21)
Wave 3	41	53.56 ± 45.32	4	139	8-251	0.85	-11.2-13.53 (1.17)
All COVID	176	62.16 ± 47.58	3	312	3-312	0.11	-16.663-1.80 (-7.43)

There was a significant increase in all-cause post-operative (PO) mortality across all three waves compared to pre-COVID time points. During Wave 1, mean PO mortality was 2.5 ± 0.71 across all quarters within the wave (p = 0.0035). During Wave 2, mean PO mortality was 1.33 ± 1.53 across all quarters within the wave (p = 0.0009). During Wave 3, mean PO mortality was 0.67 ± 0.58 across all quarters within the wave (p = 0.0060) (Table 2). There was no significant difference in 30-day PO mortality between pre-COVID and COVID time points, including comparison between waves. The average STS risk score was 4.64% overall with no significant difference between the pre-COVID-19 arm and each wave of the COVID-19 arm. 

**Table 2:** All-cause post-operative mortality stratified by each group PO: Post-operative CI: Confidence interval SD: Standard deviation

COVID	PO mortality (n)	Mean PO mortality (n)	SD (n)	P-value	CI [Mean] (n)
Pre-COVID	31	6.2	2.59		
Wave 1	5	2.5	0.71	0.0035	1.31-6.09 [3.7]
Wave 2	4	1.33	1.53	0.0009	2.15-7.59 [4.87]
Wave 3	2	0.67	0.58	0.0060	1.74-9.32 [5.53]
All COVID	18	2	2.18	0.0001	2.74-5.66 [4.20]

## Discussion

This study serves to highlight the known problem in providing care to patients with aortic stenosis: the increased time interval from diagnosis to TAVR results in greater mortality, increased hospitalization rates, and worsening functional status and quality of life [7,8]. Additionally, this study presents evidence for the critical need for timely intervention and streamlined patient care pathways for patients diagnosed with aortic stenosis [2,3]. More specifically in this study, we demonstrate the challenges of the COVID-19 pandemic across a single health system, including a significantly increased time to TAVR across Wave 1 and Wave 2 (the study’s primary outcome) and a significant increase in all-cause PO mortality across all COVID waves compared to pre-COVID time points (the study’s secondary outcome). 

We also note that our time to TAVR decreased from Wave 1 (68.44 days) to Wave 3 (53.56 days), reflective of the pre-COVID-19 arm (54.73 days). We hypothesize a few possible explanations for this: (1) our system, heart team, and our patients became more acclimated to providing and receiving medical care as the pandemic continued, (2) vaccine availability improved within our system and globally, (3) the successive COVID-19 waves had less severe global morbidity and mortality [13]. 

Patients with aortic stenosis are an inherently vulnerable population. Severe aortic stenosis that is left untreated confers a 5-year mortality >50% [14]. This markedly increased risk of death highlights the importance of early recognition of aortic stenosis, early contact with a heart team, and early treatment with TAVR when appropriate. 

Although we did demonstrate an all-cause mortality increase in our patients across all waves in our patient population, we did not see a statistically significant difference in 30-day PO mortality compared to pre-COVID data. This was possibly due to our study being underpowered and only accessing patient data within one health system. However, this did not give us the opportunity to continue to follow these patients to evaluate even longer-term differences in morbidity and mortality. This study's results underscore the importance of implementing measures to minimize TAVR procedural delays during crises like the COVID-19 pandemic, aiming to enhance patient outcomes. For example: (1) hospitals could implement a dedicated surgical unit providing care from the pre-operative phase all the way through the operating and until patients are discharged, (2) more extensive use of telemedicine, (3) preemptive planning and surge capacity management. A more extensive investigation involving larger patient cohorts and multiple healthcare systems would potentially yield a more comprehensive assessment of the long-term effects of postponed TAVR on morbidity and mortality rates. 

## Conclusions

In light of these results, we show the need for health system preparation for future pandemics and major health crises. Although difficult to ascertain the etiology or pathology of future crises, having an awareness of how these events can affect multiple patient populations indirectly and creating redundancies and mobilizing alternative healthcare access strategies is critical to limiting the known increased morbidity and mortality to our patients.

## References

[1] Edelman JJ, Thourani VH (2018). Transcatheter aortic valve replacement and surgical aortic valve replacement: both excellent therapies. J Thorac Cardiovasc Surg.

[2] Garcia S, Cubeddu RJ, Hahn RT (2021). 5-year outcomes comparing surgical versus transcatheter aortic valve replacement in patients with chronic kidney disease. JACC Cardiovasc Interv.

[3] Isogai T, Agrawal A, Shekhar S (2023). Comparison of outcomes following transcatheter aortic valve replacement requiring peripheral vascular intervention or alternative access. J Am Heart Assoc.

[4] (2022). STS/ACC TVT Registry. https://www.ncdr.com/WebNCDR/tvt/publicpage.

[5] Carroll JD, Mack MJ, Vemulapalli S (2021). STS-ACC TVT Registry of transcatheter aortic valve replacement. Ann Thorac Surg.

[6] Barbanti M, Webb JG, Gilard M, Capodanno D, Tamburino C (2017). Transcatheter aortic valve implantation in 2017: state of the art. EuroIntervention.

[7] Wijeysundera HC, Wong WW, Bennell MC, Fremes SE, Radhakrishnan S, Peterson M, Ko DT (2014). Impact of wait times on the effectiveness of transcatheter aortic valve replacement in severe aortic valve disease: a discrete event simulation model. Can J Cardiol.

[8] Elbaz-Greener G, Masih S, Fang J (2018). Temporal trends and clinical consequences of wait times for transcatheter aortic valve replacement: a population-based study. Circulation.

[9] Forman JM, Currie LM, Lauck SB, Baumbusch J (2015). Exploring changes in functional status while waiting for transcatheter aortic valve implantation. Eur J Cardiovasc Nurs.

[10] Olsson K, Näslund U, Nilsson J, Hörnsten Å (2016). Experiences of and coping with severe aortic stenosis among patients waiting for transcatheter aortic valve implantation. J Cardiovasc Nurs.

[11] Aviran E, Laks S, Benvenisti H (2020). The impact of the COVID-19 pandemic on general surgery acute admissions and urgent operations: a comparative prospective study. Isr Med Assoc J.

[12] Hunger R, König V, Stillger R, Mantke R (2022). Impact of the COVID-19 pandemic on delays in surgical procedures in Germany: a multi-center analysis of an administrative registry of 176,783 patients. Patient Saf Surg.

[13] Leidi F, Boari GE, Scarano O (2022). Comparison of the characteristics, morbidity and mortality of COVID-19 between first and second/third wave in a hospital setting in Lombardy: a retrospective cohort study. Intern Emerg Med.

[14] Strange G, Stewart S, Celermajer D (2019). Poor long-term survival in patients with moderate aortic stenosis. J Am Coll Cardiol.

